# Prevalence and clinico-morphological correlates of STK11 mutations in a large cohort of NSCLC lung adenocarcinomas

**DOI:** 10.1007/s00428-025-04392-z

**Published:** 2026-01-19

**Authors:** Rizvan Rustamov, László Füzesi, Thomas Lesser, Dagmar Täuscher, Uwe Funke, Peter Elsner, Masoud Mireskandari, Glen Kristiansen, Iver Petersen

**Affiliations:** 1 Institute of Pathology, SRH Poliklinik Gera GmbH, Gera, Germany; 2 University Hospital Jena, Jena, Germany; 3 Pathology, Medical Faculty, University of Augsburg, Augsburg, Germany; 4 Thoracic and Vascular Surgery, SRH Wald Klinikum, Gera, Germany; 5 Pneumonology/Oncology, SRH Wald Klinikum, Gera, Germany; 6 Regional Registry Unit at the Tumor Center, SRH Wald Klinikum, Gera, Germany; 7 Department of Dermatology and Allergology, SRH Wald-Klinikum, Gera, Germany; 8 Institute of Pathology, University Hospital Bonn (UKB), Venusberg-Campus 1, 53,127 Bonn, Germany

**Keywords:** STK11/LKB1, NSCLC, KRAS, TP53, Genomic profiling, Metastasis, Immunotherapy resistance, Clinicopathological features

## Abstract

STK11-mutated non–small cell lung cancers (NSCLC) show distinct clinicopathologic features, often with co-occurring KRAS and TP53 mutations that drive more aggressive disease. This study investigates the mutational landscape and clinical characteristics of STK11-mutated lung adenocarcinomas. We retrospectively reviewed 1,068 primary lung cancer cases from 2018 to 2022, identifying 14 STK11-mutant lung adenocarcinomas. We collected clinicopathologic data (demographics, histology, treatment), performed genomic profiling for co-mutations, and used immunohistochemistry to evaluate associated phenotypes. Patients with STK11 mutations (median age 67) were predominantly male smokers. KRAS or TP53 co-mutations appeared in 50% of cases. STK11/KRAS tumors showed higher metastatic rates, while STK11/TP53 tumors had increased necrosis and mitotic activity. Early-stage patients had longer survival, whereas advanced-stage cases faced significantly worse. Those treated with PD-1/PD-L1 inhibitors (Pembrolizumab) had limited benefit, with a median overall survival of 4.1 ± 2.8 months. STK11-mutant NSCLCs—especially with KRAS or TP53 co-mutations—demonstrate aggressive behavior and poor response to current immunotherapies. Comprehensive genomic profiling may help refine understanding of tumour biology and potentially inform treatment decisions. Larger studies are needed to validate these findings, but integrating genomic, pathologic, and clinical data may advance personalized therapy for these patients.

**Supplementary Information:**

The online version contains supplementary material available at 10.1007/s00428-025-04392-z.

## Introduction

The tumour suppressor gene STK11 (LKB1) encodes a serine/threonine kinase that plays a central role in regulating cellular metabolism, growth, and apoptosis. Acting upstream of AMPK, STK11 maintains energy homeostasis by inhibiting mTOR-driven anabolic processes; its loss disrupts this balance, promoting unchecked cell growth under metabolic stress [1–3]. Moreover, STK11 inactivation facilitates a metabolic shift toward glycolysis and biosynthetic pathways—hallmarks of cancer cell reprogramming [4, 5]. Beyond metabolic effects, loss of STK11 contributes to an immunosuppressive tumor microenvironment. STK11-mutant tumours typically exhibit low PD‑L1 expression, poor CD8⁺ T-cell infiltration, and elevated pro-angiogenic and immunosuppressive cytokines, impairing responsiveness to immune checkpoint inhibitors like PD-1/PD-L1 antagonists [1, 6–8].

Within lung adenocarcinoma, STK11 mutations occur in roughly 20–30% of cases and often co-occur with KRAS or TP53 mutations [1, 2, 9]. Subgroups defined by these co-mutations—especially KRAS/STK11—are marked by aggressive behaviour, including mucinous histology, lepidic growth, heightened metastatic risk, and poor prognosis [1, 2, 10, 11]. Individually, KRAS and TP53 mutations also contribute to tumor progression and therapeutic resistance [9].

Given these distinct molecular and clinicopathological features, a broader molecular characterization — including analysis of STK11 and recurrent co-mutations such as KRAS and TP53 — in combination with a consistent histopathological assessment, may help to improve understanding of tumour biology and patterns of disease progression. Such an integrated approach could ultimately support diagnostic refinement and the development of targeted therapeutic strategies, although the present study is exploratory and not powered to evaluate therapeutic implications.

In this study we aimed to delineate the mutational landscape, clinicopathological features, and treatment outcomes of STK11-mutated NSCLC by integrating genomic, pathological, and clinical data. This comprehensive analysis may help refine diagnostic characterization and contribute to hypothesis generation for future precision treatment approaches.

## Materials and methods

### Patients and samples

This retrospective study included patients with NSCLC treated at SRH Wald Klinik GmbH in Gera, Germany, between 2018 and 2022. Only cases harbouring confirmed STK11 mutations were included. Samples comprised FFPE biopsies, resected tumors, and lymphadenectomy specimens. All cases were histologically confirmed and staged according to the AJCC 8th Edition [12].

### Histopathological evaluation

Histological evaluation was performed on archived H&E- and PAS-stained slides from the original diagnostic work-up. All slides were reviewed to ensure adequate preservation and staining quality; only well-preserved sections without fading or artefacts were included.

Morphological classification and grading followed the IASLC/WHO (2021) criteria for lung adenocarcinoma. As no specific histomorphological criteria have been defined for STK11-mutated tumours, a structured semiquantitative assessment was applied to identify recurrent features within this molecular subset. Each parameter was scored on an ordinal 0–3 scale according to predefined criteria (Table S1).

The term abortive glandular pattern refers to short, incomplete, or fused glandular profiles lacking a fully developed lumen, often merging with solid areas and reflecting abortive attempts at gland formation.

The initial evaluation was performed by one of the authors during residency training and independently reviewed by two senior board-certified pathologists. Divergent interpretations were discussed and resolved by consensus. This multi-observer approach minimized inter-observer variation and ensured reproducibility of the morphological data.

In resection specimens, tumours were graded as well, moderately, or poorly differentiated, corresponding to lepidic/acinar, papillary, and solid/micropapillary predominant patterns, respectively. In small biopsies, architectural evaluation was necessarily limited; however, grading was applied using the same morphological and cytological parameters to ensure internal consistency across the cohort. These biopsy-based gradings should therefore be regarded as approximate assessments of tumour differentiation rather than formal WHO grades.

### Immunohistochemistry (IHC*)*

IHC was performed on FFPE sections using standard protocols to evaluate p53, TTF-1, Ki-67, and PD-L1 (see Table 1). All assays included appropriate controls for quality assurance.

**Table 1:** Overview of immunohistochemical staining protocols used for lung adenocarcinoma analysis

Marker	Antibody Clone	Dilution	Retrieval Buffer	pH	System Used	Manufacturer
p53	DO-7	Ready-to-use	EDTA-based buffer	8	Autostainer Link 48	DAKO (Agilent)
TTF1	8G7G3/1	Ready-to-use	Citrate buffer	9	Autostainer Link 48	DAKO (Agilent)
Ki67	MIB-1	Ready-to-use	Citrate buffer	6	Autostainer Link 48	DAKO (Agilent)
PD-L1	CAL10	1:1000	Citrate buffer	9	Vision Flex DAKO Platform	Zytomed Systems

p53 immunohistochemistry was included to correlate protein expression with TP53 mutation status determined by NGS, and to provide an internal control for tissue quality and staining consistency. Expression was assessed semiquantitatively according to established morphological patterns. Diffuse, strong nuclear staining in the majority of tumour cells (approximately ≥ 60%) was considered overexpression, whereas complete absence of staining in tumour cells with preserved internal control reactivity was classified as null pattern. A wild-type pattern was defined by variable, weak to moderate nuclear staining in a heterogeneous distribution.

The Ki-67 labelling index was assessed in areas with the highest proliferative activity (“hot spots”) by counting at least 500 tumour cell nuclei. Results were expressed as the percentage of positive nuclei. In tumours showing heterogeneous staining, evaluation was consistently performed in the most proliferative regions to ensure comparability across cases. The percentages given in Table 2 correspond to these representative hot-spot values.

**Table 2:** Trends in lung adenocarcinoma frequency and STK11-mutation prevalence at Lung-Cancer-Center-Gera, 2018–2022

	Adenocarcinoma		STK11 Mutation	
Year	Cases	%	Cases	%
2018	103	44.80%	3	2.91%
2019	94	45.40%	5	5.32%
2020	80	40.60%	3	3.75%
2021	91	44.40%	0	0%
2022	105	45.90%	3	2.86%
Overall	**473**	**44.30%**	**14**	**2.96%**

PD-L1 expression was evaluated using the tumour proportion score (TPS), defined as the percentage of viable tumour cells showing partial or complete membranous staining. Staining was performed with the CAL10 antibody clone (Zytomed Systems, Berlin, Germany), which is routinely used in our institution following internal validation against CE-IVD-approved assays and has shown reliable concordance in previous comparative evaluations. For the purposes of this study, PD-L1 results were analysed descriptively and not reinterpreted for therapeutic decision-making.

### Genomic profiling

DNA and RNA were extracted from formalin-fixed, paraffin-embedded (FFPE) tumour tissue using the Ion AmpliSeq™ Kit (Thermo Fisher Scientific, Waltham, MA). RNA was reverse-transcribed into complementary DNA (cDNA) and analysed for gene fusions involving ALK, RET, ROS1, MET, NTRK1/2/3, FGFR1/2, and PDGFRA using the Oncomine Focus Assay. DNA mutation profiling targeting STK11, KRAS, EGFR, TP53, BRAF and other recurrently altered genes in lung adenocarcinoma was performed with the Oncomine Colon and Lung Cancer Panel.

All molecular analyses were carried out externally at the Synlab Laboratory Group (Oncoscreen, MVZ Thüringen, Jena, Germany), a DAkkS-accredited reference laboratory for molecular pathology. Sequencing was performed on the Ion GeneStudio™ S5 Prime System using Ion 540/550 Chips, with template preparation on the Ion OneTouch™ 2/Ion Chef™ instruments, respectively. Both assays provide complete coverage of all coding exons and canonical splice sites of STK11 (NM_000455.5). Sequencing runs achieved high coverage across all target regions. Copy-number alterations were not routinely assessed. The applied Oncomine Colon and Lung Cancer Panel did not include KEAP1, KMT2C, SMARCA4, or MTAP; therefore, alterations in these genes could not be assessed in the present series.

### Bioinformatics and variant validation

Data processing and variant calling were performed by Synlab using Torrent Suite Software (Thermo Fisher Scientific) with the Variant Caller Plugin, followed by annotation and interpretation in Ion Reporter Software. Variants were visually verified in Integrative Genomics Viewer (IGV) and cross-checked through an in-house analysis pipeline for confirmation and consistency. Variants were classified according to the COSMIC and ClinVar databases. The same analytical workflow and reporting criteria were applied for all cases between 2018 and 2022 to ensure consistency.

### Follow-up data

Patient data were retrieved from electronic health records and included age at diagnosis, gender, smoking status, TNM stage at diagnosis, histological features, date of diagnosis, and treatment history (including chemotherapy and immunotherapy). Patients were followed until death or censored at the date of their last recorded follow-up if still alive at the end of the study period. The most recent follow-up data were available through 2024.

Progression-free survival (PFS) was defined as the interval from treatment initiation to the first documented tumor progression or death from any cause, whichever occurred first. Overall survival (OS) was calculated from the date of diagnosis to death or last follow-up. Due to these definitions, OS and follow-up duration were closely aligned, particularly in patients with short survival times.

## Results

### Patient, tumor, and treatment characteristics

Lung cancer data were collected from the institutional registry over a five-year period (2018–2022), comprising 1,068 primary lung cancer cases. Adenocarcinoma (AC) was the most common subtype, accounting for 473 cases (44.3%), followed by squamous cell carcinoma (SCC) with 346 cases (32.4%), and 239 cases (22.4%) of unspecified epithelial neoplasms [13]. The relative distribution of these subtypes remained stable across the observation period (Table 3).

**Table 3:**
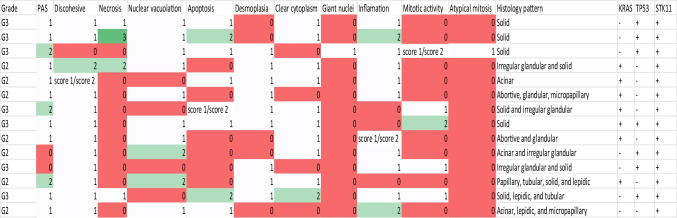
Histological characteristics of STK11-mutated lung adenocarcinomas. The table below summarizes the histological characteristics of STK11-mutated lung adenocarcinomas in our cohort. Key features were scored from 0 to 3, with 0 indicating the absence of the feature, 1 indicating mild presence, 2 indicating moderate presence, and 3 indicating strong presence

STK11 mutations were confirmed in 14 patients with AC (Table 4). The majority were male (92.9%), with a median age at diagnosis of 67 years (range: 49–86). Smoking history was reported in 78.6% of cases, while 7.1% were never-smokers and 14.3% had unknown smoking status. At the time of diagnosis, 57% of patients presented with lymph node metastases alone, and 43% with both lymphatic and distant organ metastases.

**Table 4:** Immunohistochemical and genetic characteristics of STK11-mutated lung adenocarcinoma (n = 14): a "P" symbol indicates positive expression for immunohistochemical (IHC) marker, while "N" denotes negative expression. Ki-67 values are shown as percentages, representing the proliferation index, and PD-L1 is expressed as Tumor Proportion Score (TPS) in percentages

Case No	Immunohistochemistry				Genetic		
	TTF1	p53	Ki-67	PD-L1 (TPS)	STK11	KRAS	TP53
1	N	Overexpression	50%	1%	mut	wt	mut
2	P	Overexpression	30%	0%	mut	wt	mut
3	N	Overexpression	5%	0%	mut	wt	mut
4	N	Overexpression	30%	30%	mut	mut	wt
5	P	Overexpression	20%	80%	mut	mut	wt
6	P	Wild-type	10%	15%	mut	mut	wt
7	P	Wild-type	5%	0%	mut	mut	wt
8	N	Overexpression	20%	60%	mut	mut	mut
9	P	Overexpression	10%	20%	mut	mut	wt
10	P	Null pattern*	5%	3%	mut	wt	mut
11	N	Overexpression	15%	10%	mut	wt	mut
12	P	Wild-type	5%	0%	mut	mut	wt
13	P	Overexpression	25%	10%	mut	wt	mut
14	P	Wild-type	5%	5%	mut	wt	wt

*Immunohistochemistry for p53 was completely negative, whereby P53-mutation could be verified by genetic analysis

**Fig. 1:**
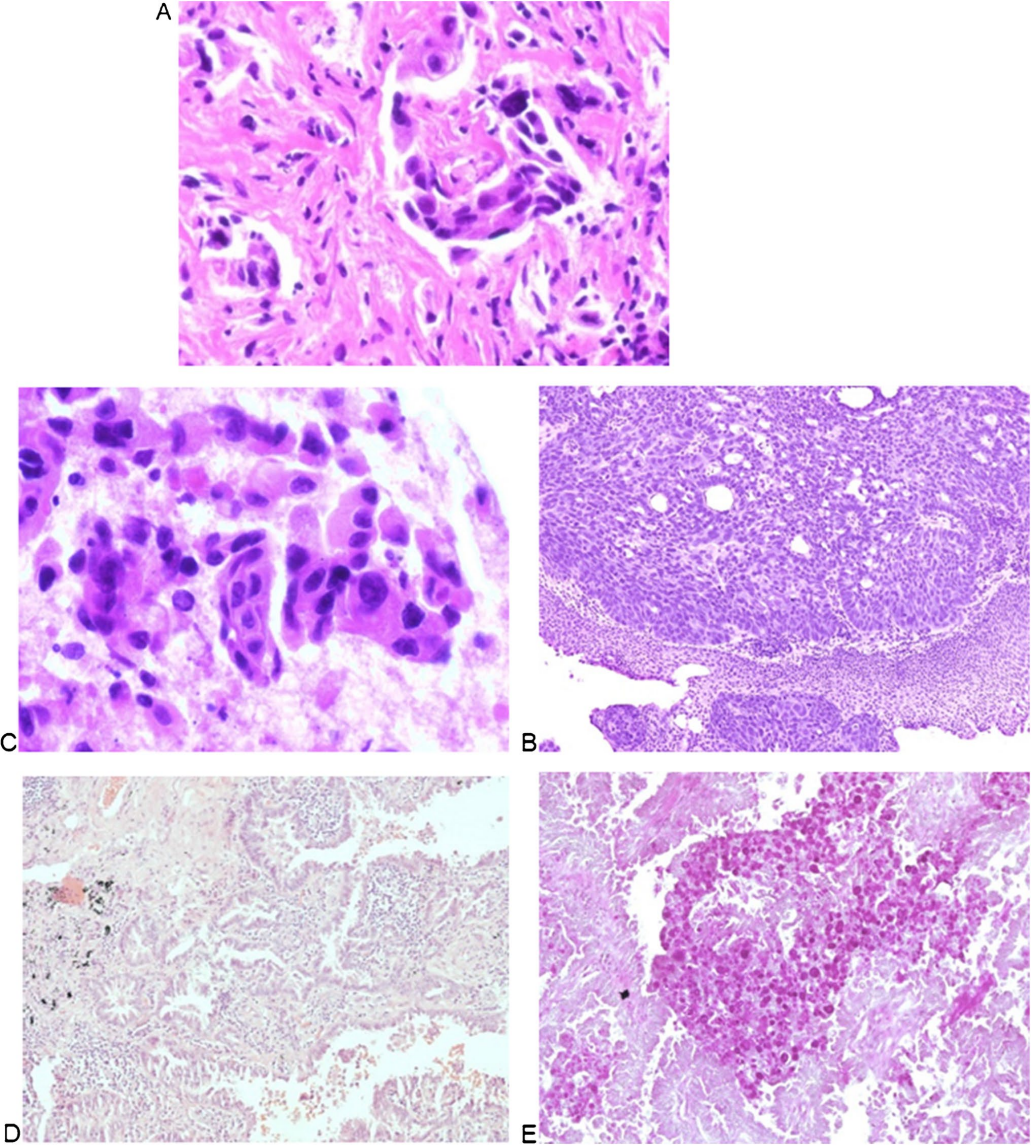

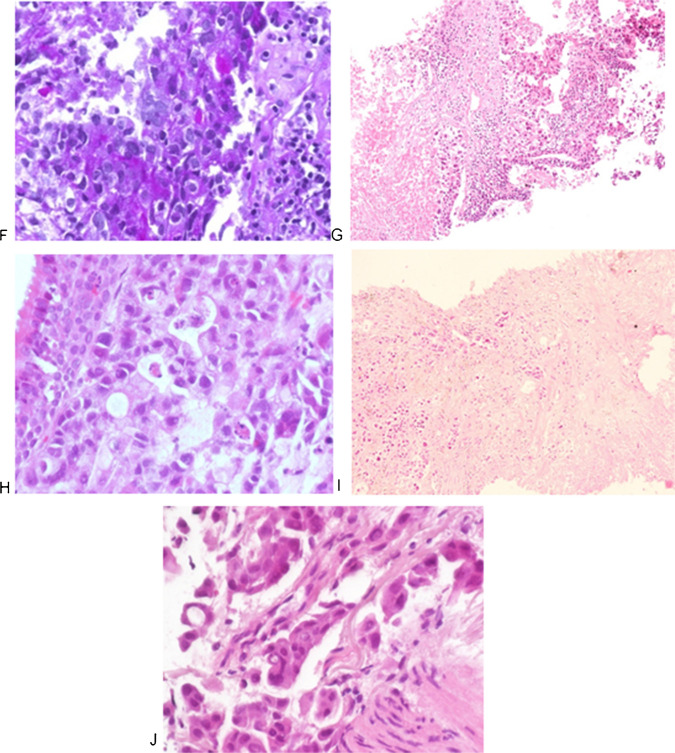
Histopathology of STK11-mutated lung adenocarcinomas: A-B) Tumor revealing pronounced hyperchromasia, anisocytosis, anisokariosis and desmoplasia (H&E, 400 × and 600x); C-D) Growth patterns: solid and lepidic (H&E, 200 × and 400x); E–F) PAS stain (400 × and 600x); G) Low-power view demonstrating extensive necrosis and inflammatory infiltration (H&E, 100x); H) Apoptotic activity with scattered apoptotic bodies (H&E, 400x); I) Areas with discohesive tumour cells lacking glandular architecture (H&E, 100x); J) Nuclear vacuolation in tumour cells (H&E, 400x)

Histological evaluation of STK11-mutated ACs revealed distinct patterns (Table 4, Fig. 1). Tumours harboring STK11 as a sole mutation predominantly showed acinar and lepidic growth, with occasional micropapillary structures. These tumours typically lacked necrosis, exhibited low nuclear vacuolation, small nuclei, and showed moderate inflammatory infiltration. In contrast, tumours with STK11 co-mutations—most commonly with KRAS or TP53—displayed more solid and glandular architecture, pronounced necrosis, marked nuclear vacuolation, and increased mitotic activity, indicating a more aggressive histological phenotype.

### Immunohistochemical findings

Immunohistochemistry revealed TTF1 positivity in 64.3% of cases. p53 expression was observed at an identical rate (64.3%), though the two markers were assessed independently, and no correlation between their expression patterns was identified, underscoring their distinct biological relevance. Notably, all TTF1-negative tumors were poorly differentiated (G3) and associated with advanced disease stages (IIB, IIIC, IVA, and IVB).

**Fig. 2:**
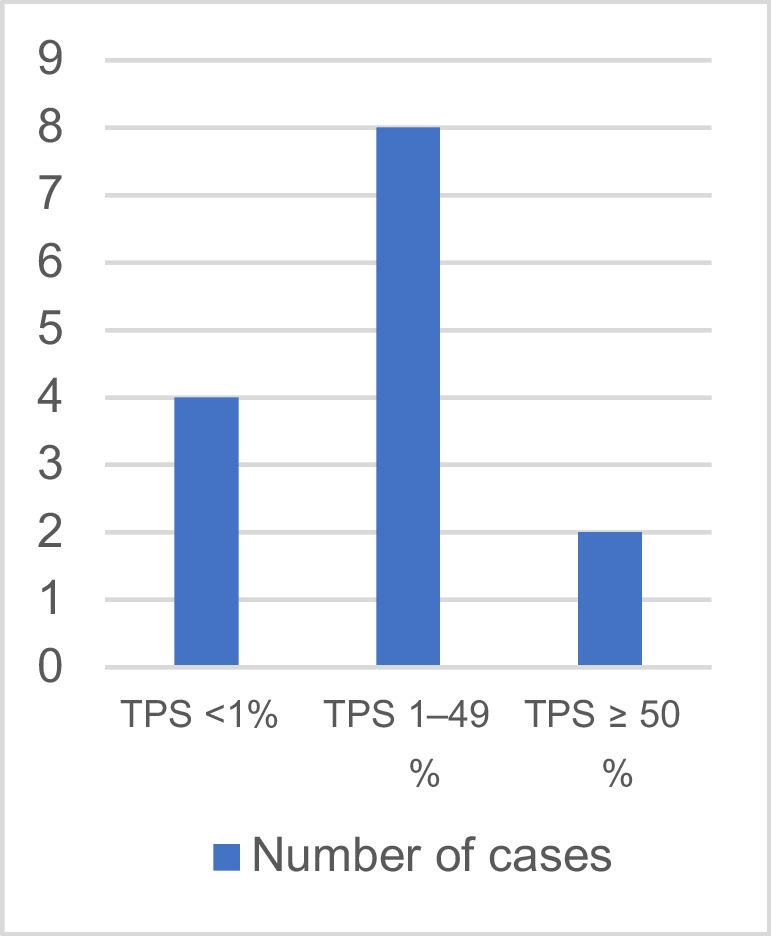
PD-L1 Tumor Proportion Score (TPS) distribution in 14 STK11-mutated lung adenocarcinoma

Ki-67 proliferation indices ranged from 5 to 50%. The PD-L1 Tumor Proportion Score (TPS) varied between 0 and 80%. In 28.6% of cases, TPS was < 1%; in 57.1%, TPS ranged from 1 to 49%; and in 14.3%, TPS was ≥ 50% (Fig. 2).

### Mutation analysis and correlation with immunohistochemistry and histology

STK11-mutated lung adenocarcinomas (ACs) displayed distinct histopathological and immunophenotypic characteristics depending on their co-mutation profiles (Tables 4 and 5). A histological continuum was observed, ranging from relatively indolent features in STK11-only tumors to highly aggressive morphologies in cases with multiple co-mutations.

**Table 5:** Mutations of STK11-, KRAS- and TP53-gene

Case No	STK11 (*n* = 14)	KRAS (*n* = 7)	TP53 (*n* = 7)
1	c.580G > T, p.D194Y		c.734G > 10, p.G245V
2	c.992_993delGGinsTTT, p.R331Lfs*29 (20%)		c.469G > T, p.V157F (10%)
3	c.824dupC, p.D277Rfs*8 (12%)		c.742C > T, p.R248W (25%)
4	c.842_843delinsT, p.P281Lfs*6 (43%)	c.34G > T, p.G12C (50%)	
5	c.993dupG, p.W332Vfs*28 (8%)	c.35G > T, p.G12V (6%)	
6	c.146_152delACCTGAT, p.Y49Wfs*13 (17%)	c.37G > T, p.G13C (20%)	
7	c.182delG, p.G61Afs*3 (16%)	c.34G > T, p.G12C (12%)	
8	c.598-1G > C, splice defect (55%)	c.34G > T, p.G12C (40%)	c.848G > A, p.R283H (77%)
9	c.179dupA, p.Y60* (5%)	c.34_35delGGinsTT, p.G12F (14%)	
10	c.784A > T, p.K262* (21%)		c.240_243delTACA, p.T81Rfs*41 (8%) + c.273G > A, p.W91* (10%)
11	c.850G > C, p.D284H (36%)		c.812A > T, p.E271V (35%)
12	c.151_162del, p.Met51_Leu54del	c.34G > T, p.Gly12Cys	
13	c.298C > T, p.Gln100*		c.845G > C, p.Arg282Pro
14	c.839C > T, p.Pro280Leu (8%, NM_000455.5)		

The tumor harboring an isolated STK11 mutation (7.1% of cases) exhibited mixed lepidic, acinar, and micropapillary growth patterns. Histologically, it showed limited mucin production (low PAS staining), mild nuclear vacuolation and apoptosis, and moderate inflammatory infiltration. Immunohistochemically, it was TTF1 positive, p53 negative, with a Ki-67 index of 5% and a PD-L1 Tumor Proportion Score (TPS) of 5%.

STK11/KRAS co-mutated tumors accounted for 42.9% of cases. KRAS mutations were primarily located at codon 12 (p.G12.). These tumors predominantly showed acinar, irregular glandular, and solid growth patterns. PAS staining was mild to moderate, with corresponding levels of nuclear vacuolation and desmoplasia. Inflammatory infiltration was generally low to moderate. Most tumors expressed TTF1, although two were negative. Ki-67 indices ranged from 5 to 30%, and PD-L1 TPS values varied widely between 0 and 80%.

Another 42.9% of tumors harbored STK11/TP53 co-mutations. These cases typically demonstrated solid architecture with extensive necrosis, mild to moderate nuclear vacuolation, occasional giant nuclei, and mild to moderate mitotic activity, including atypical mitoses. TTF1 positivity was observed in three cases. Concordance between p53 immunohistochemistry and TP53 mutation status was 71.4%; in 21.4% of cases, p53 protein was expressed despite wild-type TP53, and in 7.1%, p53 expression was absent despite an underlying TP53 mutation. Ki-67 indices ranged from 5 to 50%, and PD-L1 TPS values were uniformly low (0–10%).

The single case (7.1%) with STK11/KRAS/TP53 triple mutations exhibited the most aggressive histological profile. It showed a solid growth pattern, mild PAS positivity, moderate mitotic activity, mild to moderate nuclear vacuolation, and mild apoptosis. The tumor was TTF1 negative, p53 strongly positive, with a Ki-67 index of 20% and a PD-L1 TPS of 60%.

**Fig. 3:**
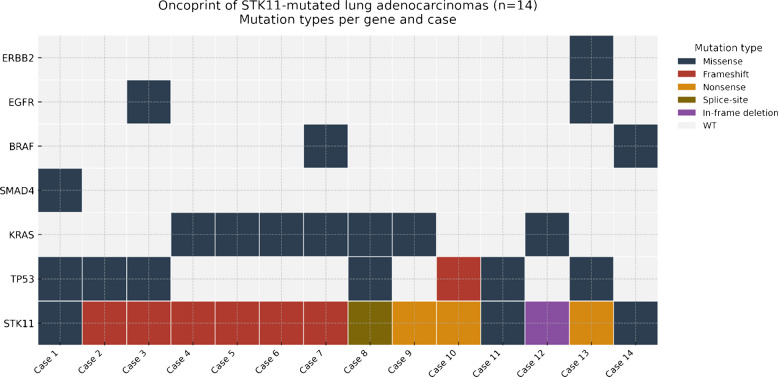
Oncoprint summarizing genomic alterations in 14 STK11-mutated lung adenocarcinomas. Each column represents one case; each row one gene. Colours indicate mutation type (missense, frameshift, nonsense, splice-site, in-frame deletion; wild type in light grey). Co-mutations in KRAS and TP53 were most frequent; additional variants in BRAF, EGFR, ERBB2, and SMAD4 occurred sporadically

Additional mutations were detected in a minority of cases, including alterations in EGFR, SMAD4, MAP2K1, and ERBB2 (each in 1 case, 7.1%) and BRAF mutations in 2 cases (14.3%). The overall mutation landscape is summarized in Fig. 3.

### Postoperative and post-therapeutic outcomes

The median overall survival (OS) for the cohort was 6.4 months (194 days), with an interquartile range (IQR) of 1.4–9.8 months. At the time of data cutoff, 28.6% of patients were alive and 71.4% were deceased (Table 6).

**Table 6:** Clinico-pathological characteristics of patients with STK11-mutated lung adenocarcinoma (n = 14)

Case No	Age	Gender	Smoking Status	Grade	Stage/UICC	Therapy	OS (in months)
1	80	M	+	G2	T1bN0M0/IA2	surgery	22*
2	67	M	+	G3	T1bN0M0/IA2	surgery + adjuvant systemic therapy	27*
3	73	M	unknown	G2	T1cN0M0/IA3	surgery + radiotherapy	48*
4	81	M	unknown	G2	T2aN0M0/IB	surgery	13*
5	80	M	+	G3	T3N0M0/IIB	surgery	9.8
6	64	M	+	G3	T3N0M0/IIB	chemotherapy	6.9
7	60	M	+	G3	T4N2M0/IIIB	surgery + chemotherapy	25.5
8	81	M	never/-	G3	T4N3M0/IIIC	chemotherapy	4.5
9	86	M	+	G2	T4N3M1a/IVA	chemotherapy + immunotherapy	1.3
10	49	M	+	G2	T4N3M1a/IVA	symptomatic	1.4
11	74	F	+	G2	T4N2M1b/IVA	symptomatic + radiotherapy of MTS	0.6
12	69	M	+	G3	T3N3M1c/IVB	chemotherapy + immunotherapy	2.3
13	68	M	+	G3	T4N3M1c/IVB	chemotherapy + immunotherapy	6.8
14	66	M	+	G2	T3N3M1c/IVB	symptomatic + radiotherapy of MTS	4.1

*denotes patients who were still alive at the time of the study

#### Stage I (28.6%)

Patients with Stage I disease experienced the most favorable outcomes, underscoring the benefit of early detection and timely surgical intervention. One patient with a T1bN0M0 (IA2) tumor underwent surgery alone and remains alive with an OS of 22 + months (STK11 +/KRAS +/TP53–). A second IA2 patient received surgery followed by adjuvant systemic therapy and achieved an OS of 27 + months (STK11 +/KRAS–/TP53 +). A third patient with a T1cN0M0 (IA3) tumor treated with surgery and postoperative radiotherapy had the longest OS in this group at 48 + months (STK11 +/KRAS–/TP53 +). A fourth patient with a T2aN0M0 (IB) tumor underwent surgery alone and remains alive with an OS of 13 + months (STK11 +/KRAS–/TP53–).

All four patients are currently alive and under active follow-up, demonstrating the effectiveness of curative surgery in early-stage STK11-mutated disease.

#### Stage II (14.3%)

Two patients with T3N0M0 (IIB) tumors showed less favorable outcomes: One underwent surgery and achieved an OS of 9.8 months (STK11 +/KRAS +/TP53–). The other received chemotherapy only and had a shorter OS of 6.9 months (STK11 +/KRAS–/TP53 +).

#### Stage III (14.3%)

Treatment strategy appeared to influence outcomes in Stage III disease: A patient with a T4N2M0 (IIIB) tumor treated with surgery and chemotherapy achieved an OS of 25.5 months (STK11 +/KRAS–/TP53 +). In contrast, a patient with a T4N3M0 (IIIC) tumor who received chemotherapy alone had an OS of 4.5 months (STK11 +/KRAS–/TP53 +).

#### Stage IV (42.9%)

Patients with Stage IV disease had the poorest prognosis. Stage IVA (3 cases)—two patients with T4N3M1a tumors had short survival despite different therapies: One received chemotherapy and immunotherapy (OS: 1.3 months; STK11 +/KRAS +/TP53–). The other was managed symptomatically (OS: 1.4 months; STK11 +/KRAS +/TP53–). A third patient with a T4N2M1b tumor treated with palliative radiotherapy had the shortest OS at 0.6 months (STK11 +/KRAS +/TP53–). Stage IVB (3 cases)—two patients received chemo-immunotherapy: one with a triple-mutated tumor (STK11 +/KRAS +/TP53 +) had an OS of 2.3 months. The other (STK11 +/KRAS–/TP53 +) achieved an OS of 6.8 months. The third patient (STK11 +/KRAS +/TP53–) received palliative radiotherapy and had an OS of 4.1 months. Pembrolizumab-based immunotherapy was administered in 3 of 6 Stage IV cases. The corresponding PD-L1 TPS values were 60%, 20%, and 10%. Despite high PD-L1 expression in some tumours, the clinical response was limited, with a mean OS of 4.07 ± 2.75 months, reflecting the limited efficacy of immune checkpoint inhibition in this molecular context.

## Discussion

Lung cancer remains the leading cause of cancer-related mortality worldwide, with approximately 1.8 million new diagnoses and nearly 1.6 million deaths annually [14, 15]. Non-small cell lung cancer (NSCLC) accounts for roughly 85% of all lung cancers [16], with adenocarcinoma (AC) representing the most common histological subtype, comprising 40–50% of NSCLC cases [17]. In Germany, lung cancer ranks as the second leading cause of death among men, following cardiovascular disease, and is responsible for 6.5% of all deaths [18].

Recent advances in molecular profiling have identified several key genetic alterations in lung cancer, including mutations in EGFR, ALK, KRAS, BRAF, and, more recently, STK11 [19]. Comprehensive genomic studies have shown that co-mutations can significantly influence the biological behavior and clinical trajectory of STK11-mutated lung AC [20]. Our study focused on this unique subgroup, which exhibits distinct clinicopathological and molecular features. Although STK11 mutations are most frequently found in lung AC, they also occur in squamous cell carcinoma and large cell carcinoma [21, 22]. Shen et al. reported a higher prevalence of STK11 mutations among Caucasians (16%) compared to Asians (4–7%) [23].

In our cohort, the prevalence of STK11 mutations in lung AC was 2.96%, which is lower than previously reported estimates ranging from 5 to 30% [24, 25]. This discrepancy may be attributed to differences in demographic and clinical characteristics. Our cohort primarily included male smokers, in line with existing literature [26, 27], and displayed a broad range of tumor stages (I–IV), reflecting the heterogeneity of STK11-mutated lung cancer [11, 28]. All STK11-mutated cases diagnosed during a defined five-year period were included, representing a consecutive, single-centre cohort analysed by a uniform NGS workflow with complete gene coverage. Hence, the relatively low mutation prevalence likely reflects demographic and regional variation, as well as biological and environmental factors influencing mutation spectra across populations [23, 29, 30]. Notably, smoking—a known driver of mutations in genes such as KRAS and TP53—was prevalent in our cohort and frequently co-occurred with STK11 mutations [31]. These findings underscore the importance of considering demographic and lifestyle factors in genetic and therapeutic studies [29].

The histological spectrum of STK11-mutated lung AC has been described in several studies [32, 33]. While Skoulidis et al. associated these tumors with mucinous histology and lepidic growth patterns [34], our findings diverged, showing predominantly solid growth with discohesive cells, necrosis, and nuclear vacuolation. Additional features included desmoplasia, clear cytoplasm, and variable inflammatory responses. Importantly, we observed that histological characteristics varied according to the co-mutation profile: STK11/KRAS co-mutated tumors commonly showed moderate to high PAS positivity, desmoplasia, and inflammatory infiltration. STK11/TP53 co-mutated tumors, by contrast, exhibited more aggressive features, including extensive necrosis, significant nuclear vacuolation, and high mitotic activity, but less frequent desmoplasia.

These observations support the notion that genetic alterations influence not only tumor biology but also morphologic phenotype.

Consistent with previous studies, we found that TTF-1 expression was absent in poorly differentiated (G3) tumors, all of which were diagnosed at advanced stages (IIB–IVB), likely reflecting tumor dedifferentiation [35]. As expected, survival tended to be longer in early-stage than in advanced-stage tumours, reflecting the general stage-related survival pattern rather than a specific molecular effect. We also noted three cases with p53 protein accumulation, as detected by immunohistochemistry, despite the absence of TP53 mutations. This phenomenon is well-documented and may be influenced by carcinogenic exposures, epigenetic changes, or post-translational mechanisms [36]. Consequently, while p53 immunohistochemistry is commonly used as a surrogate marker for TP53 mutation status, our findings suggest that it may not always accurately reflect the underlying genotype. Given the limited size of our cohort, these observations should be interpreted with caution and ideally considered in conjunction with molecular data. In this context, p53 immunohistochemistry was primarily included to validate the correlation between protein expression and TP53 mutation status, serving as an internal quality control rather than a prognostic or predictive analysis.

In line with earlier findings [30, 37–39], our study confirmed the frequent co-occurrence of STK11 with KRAS and TP53 mutations—each present in 50% of our cohort. These co-mutations have important biological implications: KRAS mutations are associated with enhanced tumor proliferation and metastatic potential [25, 40]. TP53 mutations are hallmarks of genomic instability and poor prognosis [37, 40]. In our study, patients with STK11/KRAS co-mutations had a median OS of just 5.5 months and showed a high incidence of metastasis, reflecting their aggressive clinical course [11, 40, 41]. Similarly, STK11/TP53 co-mutated tumors were associated with poor survival (median OS: 6.0 months). These findings are consistent with existing literature highlighting the synergistic effect of these mutations on tumor progression and treatment resistance [40, 42–45].

We also identified additional genetic alterations in SMAD4, BRAF, EGFR and ERBB2, though at lower frequencies. These findings suggest the presence of distinct molecular subtypes within the STK11-mutated AC population, contributing to clinical heterogeneity [19, 46]. Although alterations in KEAP1, KMT2C, SMARCA4, and MTAP were not assessed in the present study, these co-mutations are known to interact functionally with STK11 and KRAS, contributing to metabolic rewiring and immune evasion in lung AC [47]. Future studies incorporating extended sequencing panels will be needed to delineate these relationships in greater detail.

Treatment outcomes in our cohort mirrored the complexity of the underlying molecular landscape. STK11-mutated tumors are known to exhibit poor responses to immune checkpoint inhibitors, including PD-1/PD-L1 blockade [1, 48, 49]. Our findings support this observation: despite PD-L1 TPS levels as high as 60%, patients treated with Pembrolizumab showed limited benefit, with a mean OS of only 4.07 ± 2.75 months [1, 50–52]. These results reinforce previous evidence that STK11 mutations create an immunosuppressive tumor microenvironment that impairs the efficacy of immunotherapy [1, 6, 8, 11, 53]. Even in cases with high PD-L1 expression, immune checkpoint blockade did not substantially improve outcomes, highlighting a major therapeutic challenge.

Taken together, our findings underscore the need for more effective, tailored therapeutic approaches for STK11-mutated lung AC. Despite the limited cohort size, the principal strength of this study lies in its detailed, consensus-based morphologic characterization within a uniformly sequenced, real-world STK11-mutated population. Given the limited response to current immunotherapies and the aggressive behavior of co-mutated tumors, novel strategies—possibly targeting metabolic pathways, DNA damage responses, or tumor microenvironment modulators—should be explored in future studies.

## Contributions

RR and IP conceived this study and were responsible for writing and review of the manuscript, data interpretation, and review of cases. LF, TL, DT, UF, PE and MM were responsible for data collection, interpretation of data, and review of the manuscript. GK conducted the final revision of the ms.

## Supplementary Information

Below is the link to the electronic supplementary material.

ESM 1 (DOCX 15.3 KB)

## Data Availability

Data is available upon reasonable request.
