# Supplementary material for: Prevalence and clinico-morphological correlates of STK11 mutations in a large cohort of NSCLC lung adenocarcinomas
Source: Virchows Arch. 2026 Jan 19;489(1):65–77. doi: 10.1007/s00428-025-04392-z (PMC13369655; doi:10.1007/s00428-025-04392-z)
Supplement: Supplementary file 1 — (DOCX 15.3 KB) [file 428_2025_4392_MOESM1_ESM.docx]

**Supplementary Table S1.** Semiquantitative scoring criteria (0–3) applied for morphological assessment

| **Feature** | **Score 0** | **Score 1** | **Score 2** | **Score 3** |
| --- | --- | --- | --- | --- |
| Discohesive growth | None | Focal | Moderate | Diffuse |
| Necrosis | None | <10 % | 10–50 % | >50 % |
| Apoptosis | Rare | Mild | Moderate | Marked |
| Mitotic activity (per 10 HPF) | <1 | 1–3 | 4–9 | ≥10 |
| Atypical mitoses | None | 1 figure | 2–3 figures | >3 figures |
| Cytoplasmic clarity / nuclear vacuolation | None | Focal | Patchy | Diffuse |
| Desmoplasia | None | Mild | Moderate | Marked |
| Inflammatory infiltration | None | Sparse | Moderate | Dense |
| PAS-positive mucin | Negative | Focal | Patchy | Diffuse |
| Giant nuclei | None | Occasional enlarged nuclei | Multifocal occurrence | Diffuse presence of markedly enlarged nuclei |
